# Reducing ammonia emissions and nitrate leaching in wheat–maize systems by substituting chemical fertilizers with manure

**DOI:** 10.3389/fpls.2026.1761974

**Published:** 2026-03-30

**Authors:** Jun Du, Xiao-fei Wang, Yi-chang Wei, Shah Jahan Leghari, Shun-jiang Li

**Affiliations:** 1 Institute of Plant Nutrition, Agricultural Resources and Environmental Science, Henan Academy of Agricultural Sciences/Henan Key Laboratory of Agricultural Resources and Environment, Zhengzhou, Henan, China; 2 College of Resources and Environment, Henan Agricultural University, Zhengzhou, China; 3 College of Surveying and Geo-informatics, North China University of Water Resources and Electric Power, Zhengzhou, Henan, China; 4 College of Mechanical and Electronical Engineering, Northwest A&F University, Yangling, Shaanxi, China; 5 Chinese Academy of Environmental Planning, Beijing, China

**Keywords:** ammonia measurements, nitrate leaching, chemical fertilizer, manure, wheat-maize rotation

## Abstract

**Introduction:**

Ammonia (NH_3_) emissions and nitrate (NO_3_^-^) leaching from crop fields are major contributors to non-point source environmental pollution.

**Methods:**

To optimize the resource utilization of pig manure and reduce gaseous and liquid nitrogen losses in wheat–maize rotation systems, five treatments were investigated, including 15% (OF_15_), 30% (OF_30_), and 45% (OF_45_) substitution of chemical fertilizer nitrogen with pig manure as well as a no-nitrogen control (CK) and local farmer’s fertilization method (CF). Ventilation-type NH_3_ capturing devices and NO_3_^-^ leaching lysimeters were used to systematically investigate the nitrogen (N) dynamics in the wheat–maize rotation systems from 2022 to 2024.

**Results:**

The pig manure substitution ratio had profound impacts on NH_3_ emissions, NO_3_^-^ leaching, and crop yields. When the substitution ratio of pig manure was 30%, wheat produced the highest average yield at 9,181 kg ha^-1^, with 0.256 g kg^-1^ lower NH_3_ emissions per unit of grain output compared to chemical fertilization alone. Similarly, the maize yield was 10,897 kg ha^-1^, with 0.17 lower NH_3_ emissions per unit of grain.

**Discussion:**

Comprehensive analysis suggests that 30% pig manure substitution ratio in OF_30_ will be optimal to achieve environmental and yield benefits simultaneously.

## 1 Introduction

Agricultural intensification has resulted in excessive chemical fertilization in cropping systems worldwide (Adalibieke et al., 2023; McLaughlin et al., 2024). In China, the N application rate per unit of arable land significantly exceeds the internationally recognized safety threshold (Wang et al., 2023a), yet NUE remains below 35% (Chen et al., 2025a). This “high-input, low-efficiency” fertilization pattern is particularly common in major wheat–maize production areas of the North China Plain (NCP) (Shen et al., 2025), where the N application rates typically surpass the actual crop requirement (Meng et al., 2024; Leghari et al., 2026). Consequently, there is a substantial amount of N losses through gaseous form and liquid leaching (Li et al., 2024). It triggered multidimensional environmental problems, including soil, water body, and air quality degradation (Wang et al., 2023b; Liu et al., 2024; Khan et al., 2025). Compared to the 1990s, the atmospheric NH_3_ concentrations have risen markedly (Yue et al., 2022). Similarly, groundwater pollution from NO_3_^-^ is a serious concern in different agricultural zones (Zhai et al., 2017; Chadwick et al., 2015).

The application of livestock manure in field crops can reduce the use of synthetic N fertilizer inputs and alleviate environmental degradation. Manure substitution in wheat–maize systems improves yields and decreases N losses. It has the potential to reduce NO_3_^-^ leaching by 46%, with up to 22% lower NH_3_ volatilization, while sustaining the crop yields (Guo et al., 2020). Compared to different manures, pig manure has the highest soil N availability (Xu et al., 2022). Moreover, a slow-release effect of organic N manure was formed through mineralization–immobilization cycles (He et al., 2023a; He et al., 2023b; Sun et al., 2025). It not only lessens plow pan compaction caused by long-term rotary tillage but also reduces soil urease activity through its humic acid components, thereby inhibiting the key process of NH_3_ volatilization (Zhang et al., 2023). However, organic substitution cannot be overlooked (Hou et al., 2025). Excessive application leads to C/N ratio imbalance (Zhang et al., 2025a) and causes high CH_4_ emissions. When pig manure substitution exceeds 40%, the NO_3_^-^ concentration in soil leachate surges by 280% (Shao, 2024). Meanwhile, long-term field experiments show that high-rate pig manure application increases NO_3_^-^ leaching compared to conventional fertilization during the maize growing season (Ma et al., 2024; Shao, 2024). At the same time, Xu et al. (2024a) suggest 50% application of pig manure as the best ratio to achieve environmental and yield benefits simultaneously. In contrast, Tong et al. (2023) found that manure substitutions should not exceed 25% in the wheat–maize ration system.

Previous research shows that soil, crop type, or seasonal factors influence the optimal rate of manure substitution ratio. Therefore, the conclusions in the existing literature are not uniform, highlighting a critical knowledge gap that forms the central rationale for this study (Ren et al., 2021; Wang et al., 2024b; Xu et al., 2024; Han et al., 2025; Niu et al., 2025; Yu et al., 2025). Moreover, the annual wheat–maize systems exhibit significant N depletion dynamics and larger trade-offs between crop production and emissions under organic material inputs (Chen et al., 2025b). Which pig manure ratio is optimal is still unclear based on quantitative measurement of environmental fertilizer loss. Therefore, this study aimed to optimize the resource utilization of pig manure and reduce gaseous and liquid N losses in farmlands.

## 2 Materials and methods

### 2.1 Experimental site

The experimental site is located in Qian’an village, Hua county of Henan province (35°38′0.91″N, 114°43′60″ E). The area is in the central-western part of the North China Plain. The cropping system is mainly wheat–maize rotation (Yang et al., 2025; Zhang et al., 2025b). This region has a temperate continental monsoon climate, with an annual average temperature of 13.7°C. The average precipitation is 634.3 mm (Chen et al., 2024), with high rainfall mainly concentrated from July to September (Wang et al., 2025). The soil type is loamy (USDA classification), and the basic soil properties of the cultivated layer (0–20 cm) in the experimental field are as follows: pH 8.55, OM content 12.1 g kg^-1^, NH_3_ content 3.13 mg kg^-1^, NO_3_^-^ concentration 12.26 mg kg^-1^, total N 0.73 g kg^-1^, available P content 41.6 mg kg^-1^, and K 77.3 mg kg^-1^.

### 2.2 Experimental design

There were five treatments, including CK (0 N fertilizer, single application of P and K fertilizers), CF (local farmer’s fertilization method with 270 kg N ha^-1^), OF_15_, OF_30_, and OF_45_ (15%, 30%, and 45% substitution of chemical fertilizer N with pig manure). Thus, the OF_15_, OF_30_, and OF_45_ treatments received 230, 189, and 149 kg chemical N kg ha^-1^, respectively, in the wheat field along with equivalent P and K fertilizers. In the maize field, the NPK fertilizers were applied at an equal rate without the addition of pig manure, except for the CK treatment, which received zero application of chemical N fertilizer. The plots were 6 m × 4 m = 24 m² with three replications; each treatment was arranged randomly. Pig manure was applied once during wheat base fertilization. The fertilization methods were broadcasting and plowing in the wheat crop, whereas maize fertilization involved furrow burial and broadcasting. The specific application protocols are detailed in **Table 1**. The pig manure was provided by Shuaxian Muyuan 13th Branch, a large-scale, standardized livestock operation in China. The physicochemical indexes of the test pig manure were as follows: OM content, N, P_2_O_5_, and K_2_O were 60.3%, 2.9%, 4.2%, and 0.7%, respectively. The field experiment was conducted on a wheat–maize rotation system from 2022 to 2024. The test wheat variety was Zhengmai 1860, and the maize variety was Zhengdan 958. The sowing date of wheat was October 16–18 and June 2–8 June. There were six irrigation frequencies in wheat and three in maize.

**Table 1:** Fertilization of each treatment.

Treatments	Wheat season						Maize season			
	Urea N (kg ha^-1^)		Pig manure N		Total P (P_2_O_5_)	Total K (K_2_O)	Urea N (kg hm^-2^)		Total P (P_2_O_5_)	Total K (K_2_O)
	Base-N	Top-N	⇄	kg ha^-1^	kg ha^-1^	kg ha^-1^	Base-N	Top-N	kg ha^-1^	kg ha^-1^
CK	0	0	0%	0	120	120	0	0	90	120
CF	162	108	0%	0	120	120	108	162	90	120
OF_15_	121.5	108	15%	40.5	120	120	108	162	90	120
OF_30_	81	108	30%	81	120	120	108	162	90	120
OF_45_	40.5	108	45%	121.5	120	120	108	162	90	120

⇄ is replacement of chemical N fertilizer with organic manure. CK is no N fertilizer, single application of P and K fertilizers, CF is local farmer’s fertilization method, OF_15_ is 15% substitution of chemical fertilizer N with pig manure during wheat base fertilization, OF_30_ is 30% substitution, and OF_45_ is 45% substitution.

### 2.3 NH_3_ volatilization sample collection and determination

**Figure 1:**
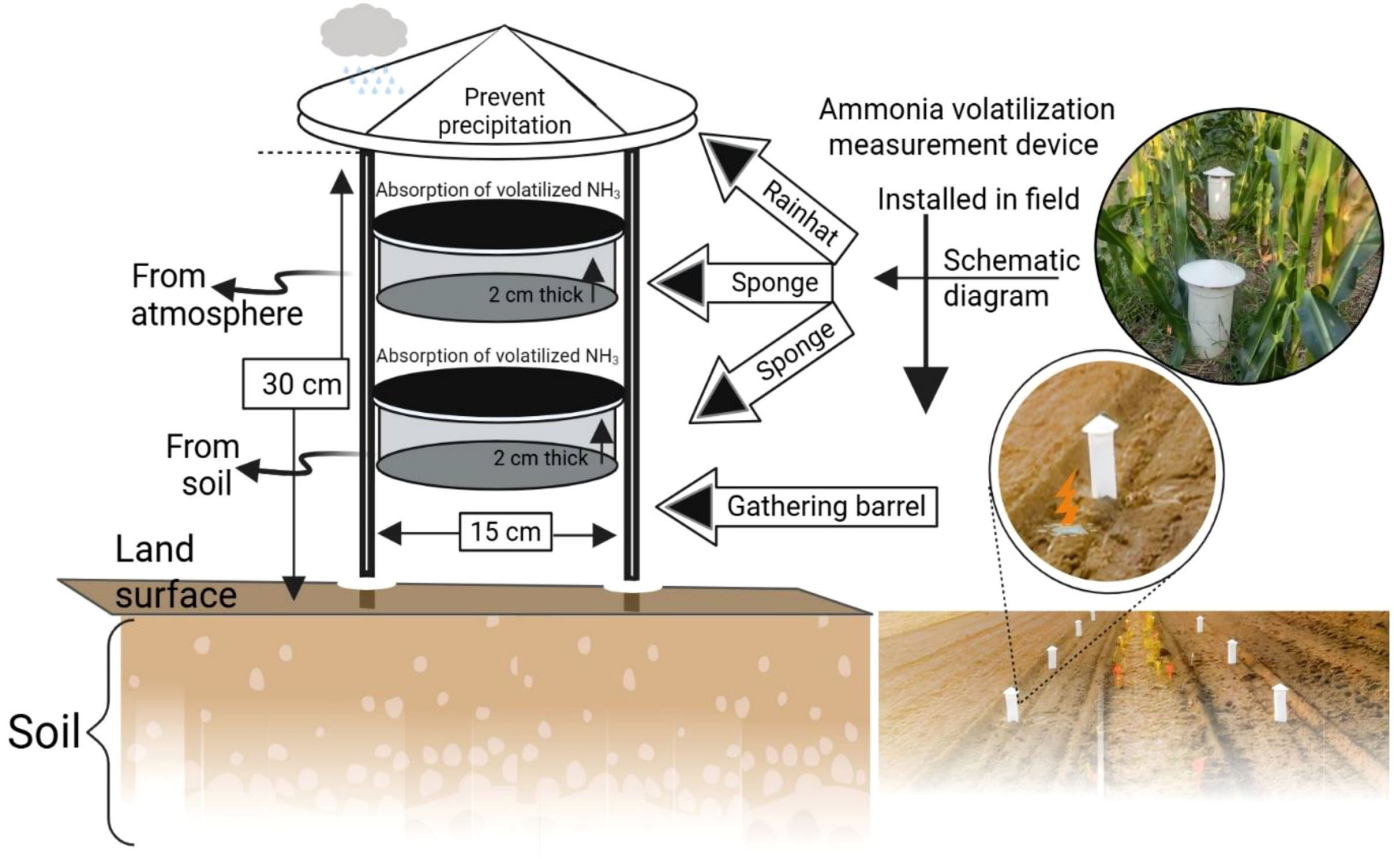
Schematic diagram of the NH_3_ volatilization collection device.

Soil NH_3_ volatilization was measured using the sponge aeration method. The NH_3_ collection device is shown in **Figure 1**. The device was inserted 5 cm into the soil. Two 2-cm-thick, 16-cm-diameter sponges were soaked in 30 mL of glycerol phosphate solution (200 mL phosphoric acid + 40 mL glycerol, diluted to 1000 mL with deionized water) and placed at the middle and top of the device, respectively. The middle sponge absorbed volatilized NH_3_ from the soil, while the top sponge isolated atmospheric NH_3_. The top sponge was replaced every 2 days. Samples were taken at 9:00 a.m. The middle sponge was removed, placed into numbered sealed bags, and then transported to the laboratory for the extraction of absorbed NH_3_ using potassium chloride solution and further analysis. The sampling frequency was as follows: once every 5 days after wheat fertilization (first 5 days) and, later, once every 2 days. Similarly, it was also once every 5 days after maize fertilization (first 4 days) and once every 2 days.

### 2.4 Collection and determination of NO_3_^-^ leaching solution

**Figure 2:**
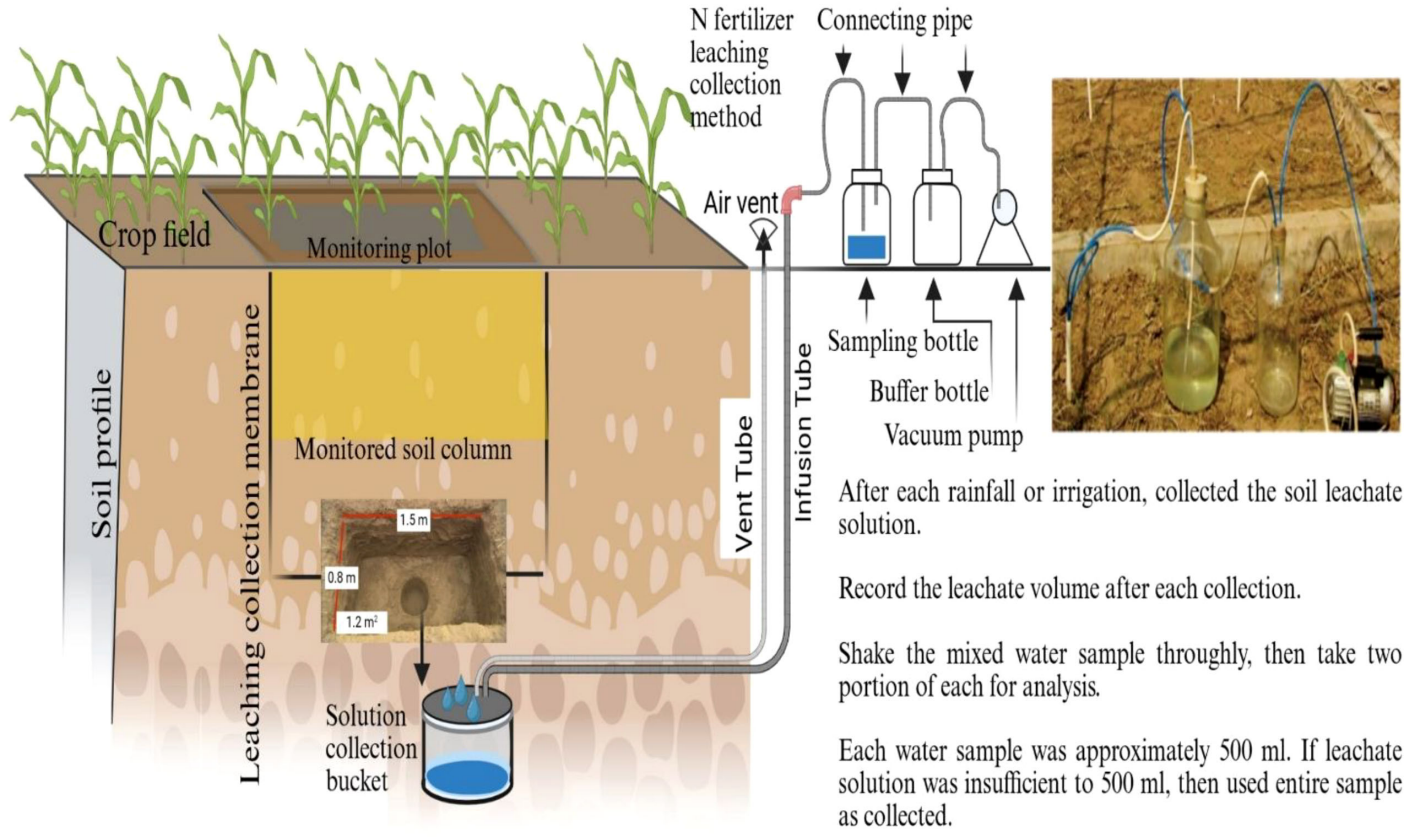
Schematic diagram of field NO_3_^-^ measurement in a crop field.

The collection of leachate samples followed the methods for measuring soil leaching and sample collection in farmlands as outlined in “Methods and Practices for Monitoring Non-point Source Pollution in Farmland” (Liu et al., 2021) with minor modifications (**Figure 2**). After each rainfall event, soil leachate NO_3_^-^ was collected using a field infiltration tank. The leachate volume was recorded after each rainfall event. Two mixed water samples were obtained by shaking the leachate, one for analysis and another as backup. Each water sample was approximately 500 mL, if less than 500 mL was collected. NO_3_^-^ content in the leachate was determined using a continuous flow analyzer.

### 2.5 Measurement of crop yield and soil samples

After crop maturity, wheat and maize plots were harvested (3 m² for wheat and 8 m² for maize). The crops were threshed, sun-dried, and weighed to calculate yields. In October 2024, following the maize harvest, a five-point sampling method was employed. The soil samples were collected at intervals of 20-cm depth using a soil drill, mixed thoroughly, and placed in a self-sealing bag. The samples were extracted with a 2.0-mol/L potassium chloride solution. Following oscillatory filtration, soil NH_4_^+^ and NO_3_^-^ contents were analyzed using a flow injection analyzer.

### 2.6 Calculation of NH_3_ volatilization and NO_3_^-^ losses

The calculation formula for soil NH_3_ volatilization rate (F, kg ha^−2^ d^−1^) is as follows (Equation 1):

(1) F=C×V×10000/(T×A)

where *C* represents the concentration of NH_3_ measured by the flow analyzer (mg/L), *V* denotes the extraction volume (L), _T_ indicates the cumulative time (d), and *A* stands for the cross-sectional area of the circular PVC-tube (m²).

Accumulated ammonia volatiles (P) (Equation 2)

(2) $$P=\sum F_{1}\times D_{1}+F_{2}\times D_{2}+\ldots \ldots +F_{n}\times D_{n}$$

where Fn is volatilization rate of soil NH_3_ at the *n*-th collection time, and *Dn* is the number of days between the last time of the *n*-th collection.

Ammonia volatilization coefficient (η) (Equation 3)

(3) η(%)=(Pt−Pck) /Nw×100

where *Ƞ* is NH_3_ emission coefficient (%), *Pt* is NH_3_ volatilization of the fertilized treatment (g), *Pck* is NH_3_ volatilization of the control area (g), and *Nw* is N application amount.

Emissions of ammonia per unit of output (Equation 4)

(4) $$W^{N}H_{3}(g\cdot kg^{-1})=P/Y\times 100\%$$

where *^W^NH_3_* is volatile amount of NH_3_ per unit yield (g kg^−1^), *P* is cumulative volatile amount of NH_3_ (g), and *Y* is the crop yield.

Nitrate nitrogen leaching amount (P, kg·ha^−1^) (Equation 5)

(5) $$P=\sum_{n}V_{\text{n}}\times C_{n}/S\times k$$

where *P* is the leaching amount of NO_3_^-^ (kg ha^−1^), *Vn* is the volume of leachate collected for the *n*-th time (L), *Cn* is the concentration of NO_3_^-^ in the leach solution for the *n*-th time (mg L^−1^), *S* is the area of the monitoring unit (1.5 m^2^), and *k* is the conversion coefficient from the monitoring unit to hectares.

Nitrogen state nitrogen apparent leaching coefficient, *µ* (%) (Equation 6)

(6) $$µ(\%)=(L_{t}-L_{ck})/Nw\times 100$$

where *µ* is the apparent leaching coefficient of NO_3_^-^ (%), *Lt* is the leaching amount of NO_3_^-^ in the N fertilizer treatment, *Lck* is the leaching amount of NO_3_^-^ in the non-N fertilizer treatment, and *Nw* is the amount of nitrogen applied.

### 2.7 Statistical analysis and software

Data processing and statistical analysis (ANOVA) were performed using SPSS software to determine the effects of pig manure substitution ratios on wheat–maize yields and N dynamics compared to conventional chemical fertilizer alone. The graphs were prepared using Origin 2025b. A schematic diagram of the NH_3_ volatilization collection device and NO_3_^-^ was drawn using biorender.com.

## 3 Results

### 3.1 Emissions of NH_3_ during wheat and maize growth progress

**Figure 3:**
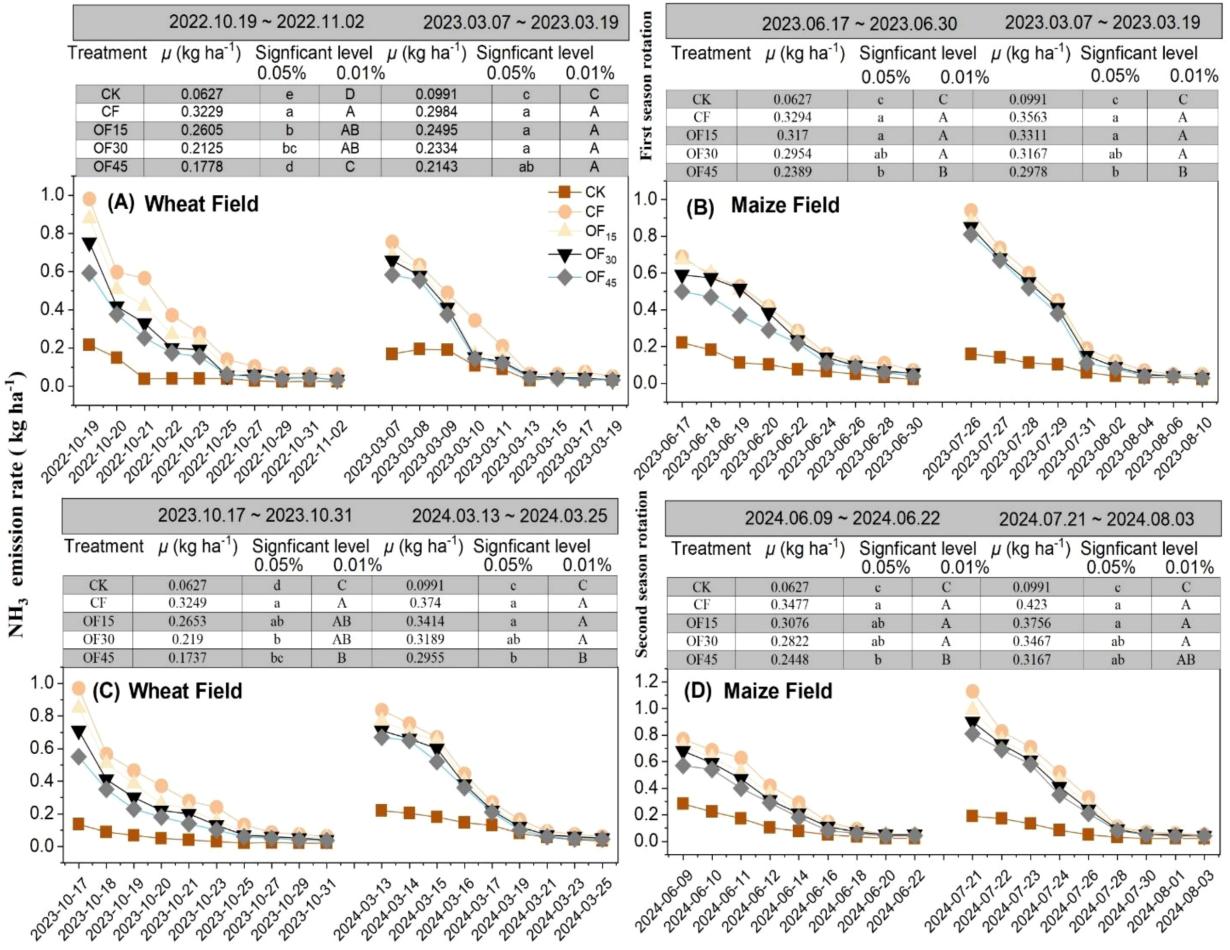
Characteristics of NH_3_ volatilization rate changes in wheat–maize rotation systems under different fertilization treatments. **(A–D)** CK is no N fertilizer, single application of P and K fertilizers, CF is local farmer’s fertilization method, OF_15_ is 15% substitution of chemical fertilizer N with pig manure during wheat base fertilization, OF_30_ is 30% substitution, and OF_45_ is 45% substitution.

The dynamic changes of NH_3_ emissions in wheat–maize rotation systems are visualized in **Figure 3**. NH_3_ emissions in all treatment groups fluctuated with pig manure application rates. Initially, there were higher NH_3_ emissions and they gradually declined in both wheat and maize fields. After fertilization in the wheat field, all treatments experienced rapid increases in NH_3_ emissions. During the first growing season (2022–2023), the chemical fertilizer treatment (0% pig manure) reached the highest NH_3_ gaseous loss at 0.32 kg ha^−1^ d^−1^ on average. However, when the pig manure ratio increased to 30%–45% (OF_45_ and OF_45_), the peak value dropped significantly to 0.18–0.21 kg ha^−1^ d^−1^ (*p* < 0.05) at the early growth stages. At the late growth stage, the mean NH_3_ emissions were also significantly lower in OF_30_ and OF_45_ than in CF by 23%–30%, indicating that pig manure amendment effectively suppressed NH_3_ emissions. The average NH_3_ volatilization was 0.30, 0.25, 0.23, and 0.21 kg ha^−1^ d^−1^ in the CF, OF_15_, OF_30_, and OF_45_ treatments, respectively. The wheat field in the second rotation (2023–2024) had average NH_3_ emissions of 0.08, 0.35, 0.30, 0.27, and 0.23 kg ha^−1^ d^−1^ in the CK, CF, OF_15_, OF_30_, and OF_45_ treatments, respectively. Regarding NH_3_ emissions in the maize field, the OF_15_ treatment had peak NH_3_ emissions at the initial stage, reaching 0.71 kg m^−2^ d^−1^, followed by a gradual decline to 0.04 kg m^−2^ d^−1^. The OF_45_ treatment exhibited lower average NH_3_ emissions, with a maximum value of 0.55 kg m^−2^ d^−1^, which stabilized at 0.04 kg m^−2^ d^−1^ by the end. Compared to the non-substitution treatment (control group), increasing the pig manure substitution ratio led to an overall reduction in NH_3_ rates, which was likely due to the slow N release from organic materials. Overall, the OF_30_ and OF_45_ treatments indicated a decreasing trend in NH_3_ volatilization rates with higher substitution ratios, particularly during the mid-growth stage of maize, where the decrease was more pronounced. In addition, the cumulative NH_3_ volatilization amounts varied among treatments, with the annual cumulative value for the OF_45_ treatment being approximately 20% lower than that of the OF_30_ treatment, demonstrating the positive effect of pig manure substitution in reducing ammonia emissions from farmlands.

### 3.2 Leaching of NO_3_^-^ during wheat and maize growth progress

**Figure 4:**
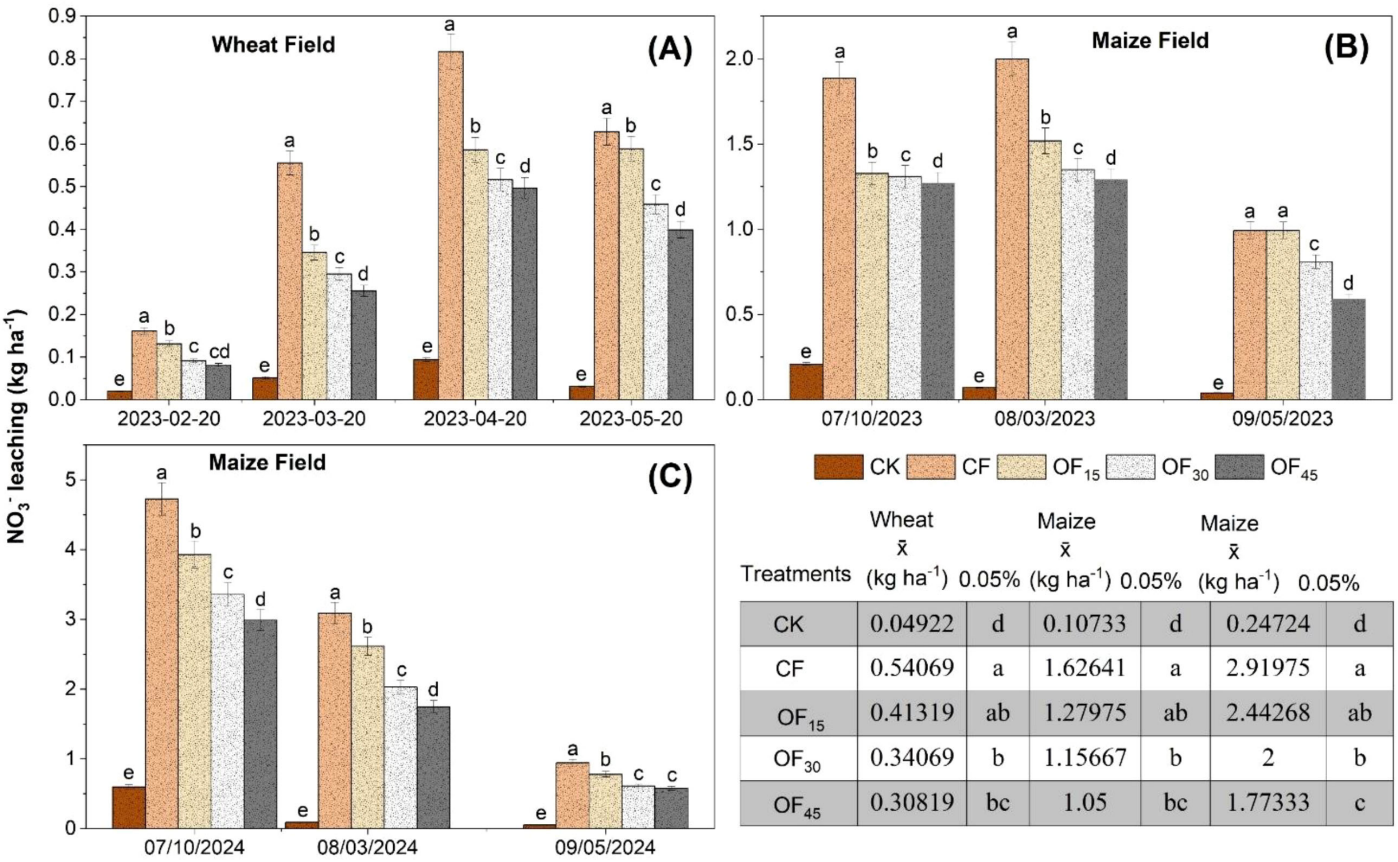
The leaching amount of NO_3-_ in soil during the wheat **(A)** and maize **(B, C)** rotation cycle from 2022 to 2024 (there was no leaching due to less rainfall in the wheat season in 2024). CK is no N fertilizer, single application of P and K fertilizers, CF is local farmer's fertilization method, OF_15_ is 15% substitution of chemical fertilizer N with pig manure during wheat base fertilization, OF_30_ is 30% substitution, and OF_45_ is 45% substitution.

NO_3_^-^ leaching at different time intervals of crop growth progress is shown in **Figure 4**. There was no NO_3_^-^ leaching in the second growing season of wheat (October 2023–2024) due to low rainfall. In the first wheat growing season (2022–2023), the CF treatment resulted in the highest NO_3_^-^ leaching loss by 0.54 kg ha^−1^ on average. All pig manure fertilization ratios reduced NO_3_^-^ leaching, with the reduction magnitude directly corresponding to the application rate. The average NO_3_^-^ leaching rates were 0.41, 0.34, and 0.31 kg ha^−1^ in January, February, March, April, and May under the OF_15_, OF_30_, and OF_45_ treatments, respectively. Thus, OF_15_ decreased NO_3_^-^ leaching by 24%, OF_30_ by 37%, and OF_45_ by 43% compared to the CF of conventional chemical N fertilization alone. Considering maize growth progress, OF_15_, OF30, and OF45 pig manure ratios decreased NO_3_^-^ leaching by 22%, 29%, and 37%, respectively, compared to CF in the first season (2023). In subsequent rotation, these pig manure incorporation rates decreased NO_3_^-^ leaching by 16%, 32%, and 39%, indicating that pig manure application mitigated fertilizer leaching losses effectively across the two growing seasons.

### 3.3 Dynamic changes in soil N concentrations during wheat and maize growth progress

**Figure 5:**
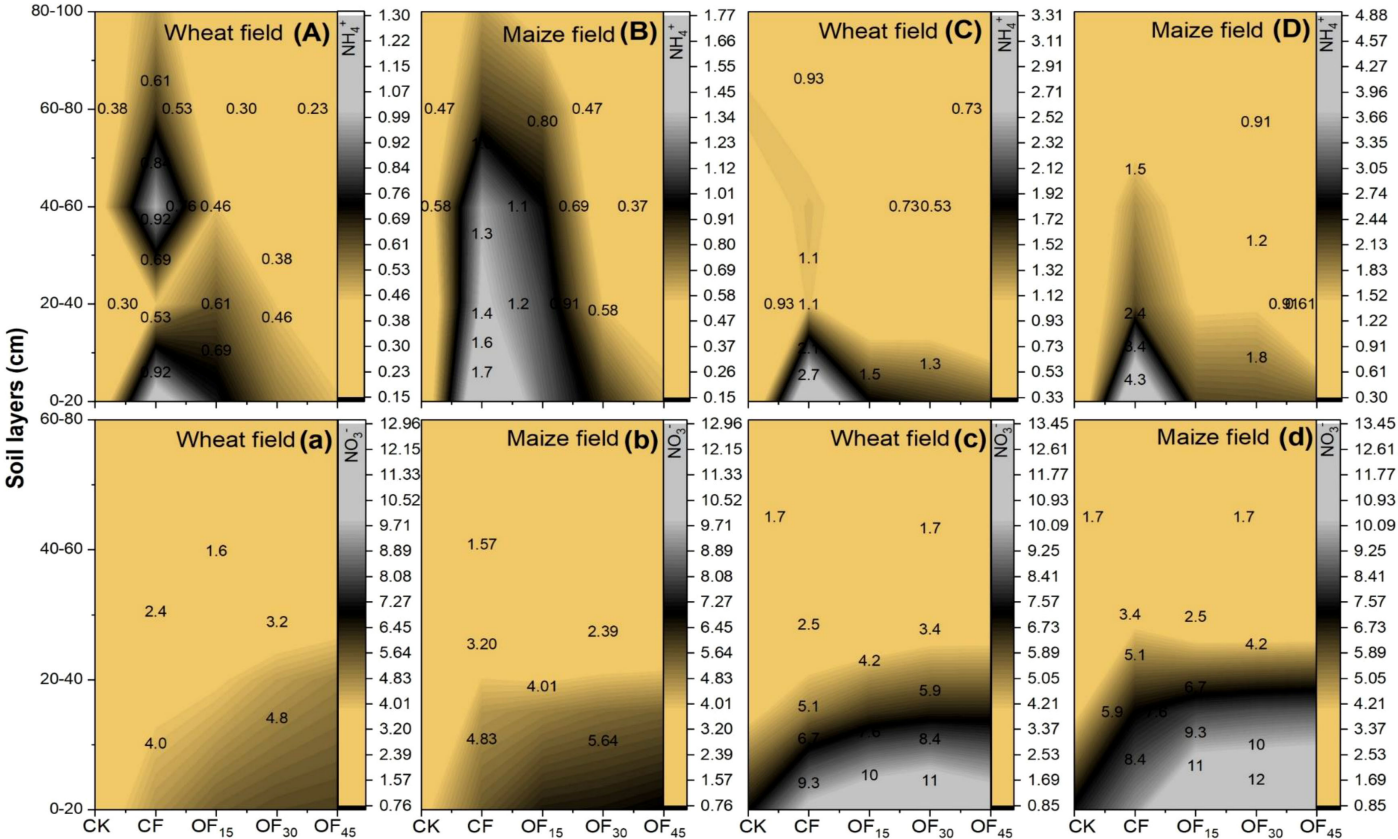
Measured values of soil NH_4_^+^ and NO_3_^-^ (mg kg^-1^) in different soil layers. Capital **(A, B)** and small-letter **(a, b)** represent first season measurements. Similarly, capital **(C, D)** and small-letter **(c, d)** represent last year’s data. CK is no N fertilizer, single application of P and K fertilizers, CF is local farmer’s fertilization method, OF_15_ is 15% substitution of chemical fertilizer N with pig manure during wheat base fertilization, OF_30_ is 30% substitution, and OF_45_ is 45% substitution.

The spatial distributions of NH_4_^+^ and NO_3_^-^ in different soil layers are shown in **Figure 5**. At the end of the experiment, the pig manure substitution demonstrated significant effects on soil nutrient composition. OF_15_ OF_30_, and OF_45_ decreased the soil NH_4_^+^ levels by 32%–35%, 43%–50%, and 49%–61% compared to the CF treatment in wheat field during the first (2022) and last season (2024) of the experiment. In the maize field, the pig manure also decreases NH_4_^+^ concentration by 24%–40%, 42%–58%, and 54%–68% from the 0–100-cm layers, while pig manure treatments enhanced NO_3_^-^ concentration as the amendment ratio increased. OF_15_ increased NO_3_^-^ concentrations by 7%–12%, OF_30_ by 11%–22%, and OF_45_ by 6%–27% in the wheat field.

### 3.4 Annual cumulative NH_3_ emissions and crop yields

**Figure 6:**
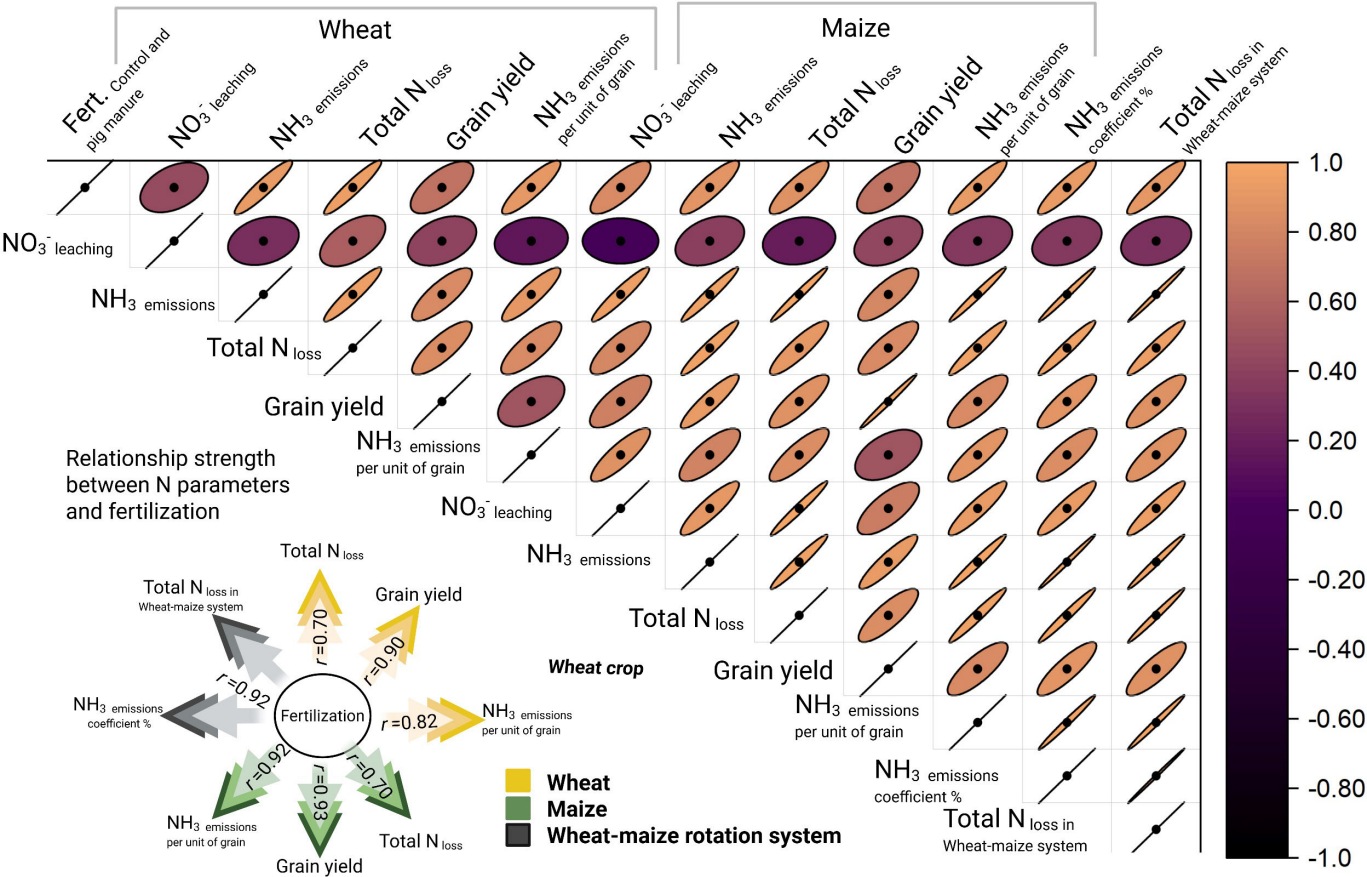
Correlation between N dynamics and fertilization, including different pig-manure substituting chemical fertilizer ratios in wheat field, maize field, and whole wheat–maize rotation systems.

From 2022 to 2024, significant differences were observed in the cumulative NH_3_ emissions, crop yields, and emission coefficients under different pig manure substitution ratios during the wheat–maize rotation cycle (**Table 2**). Specifically, under the OF_30_ treatment, the average cumulative NH_3_ emission was 4.63 kg N ha^−1^, with a crop yield of 9,123 kg ha^−1^ and an NH_3_ emission per unit yield of 0.51 kg kg^−1^. In contrast, the OF_45_ treatment resulted in a reduction of cumulative NH_3_ emissions to 4.08 kg N ha^−1^ while maintaining a crop yield of 8,268 kg ha^−1^ and an NH_3_ emission per unit yield of 0.49 kg kg^−1^. These data integrate monitoring results from both the wheat and maize seasons, demonstrating that increasing the pig manure substitution ratio helps reduce NH_3_ volatilization losses while stabilizing crop yields. Furthermore, combined analysis with NO_3_^-^ leaching data indicates that higher pig manure substitution ratios offer greater advantages in mitigating nitrogen-related environmental impacts. The correlation between fertilization and N dynamics is shown in **Figure 6**.

**Table 2:** Summary of NO_3_^-^ leaching, NH_3_ emissions, and yields of wheat–maize rotation systems.

Period	Treatment	Wheat season						Maize season				NH_3_ emission coefficient %	Σ N loss in wheat–maize kg ha^-1^
		NO_3_^-^ leaching	NH_3_ emissions	Total N loss	Grain yield	Emissions per unit of output	NO_3_^-^ leaching	NH_3_ emissions	Total N loss	Grain yield	Emissions per unit of output		
		kg ha^-1^				g kg^-1^	kg ha^-1^				g kg^-1^		
2022–2023	CK	0.2	1.8	2	5,388	0.33	0.32	2.01	2.33	6,735	0.3	—	4.33
	CF	2.16	6.6	8.76	8,696	0.76	4.88	7.39	12.28	10,127	0.73	1.89	21.04
	OF_15_	1.65	5.34	6.99	8,822	0.61	3.84	6.89	10.73	10,640	0.65	1.56	17.72
	OF_30_	1.4	4.63	6.03	9,123	0.51	3.47	6.46	9.93	10,929	0.59	1.35	15.96
	OF_45_	1.23	4.08	5.31	8,268	0.49	3.15	5.65	8.8	9,786	0.58	1.1	14.11
2023–2024	CK	0	1.92	1.92	3,527	0.54	0.74	2.05	2.8	5,768	0.36	—	4.71
	CF	0	7.6	7.6	8,617	0.88	8.76	8.18	16.94	10,080	0.81	2.19	24.55
	OF_15_	0	6.45	6.45	8,755	0.74	7.33	7.21	14.54	10,198	0.71	1.79	20.99
	OF_30_	0	5.71	5.71	9,239	0.62	6	6.62	12.62	10,865	0.61	1.55	18.33
	OF_45_	0	4.93	4.93	8,591	0.57	5.32	5.88	11.2	9,930	0.59	1.27	16.13
Annual average	CK	0.1	1.86	1.96	4,458	0.44	0.53	2.03	2.57	6,252	0.33	—	4.52
	CF	1.08	7.1	8.18	8,657	0.82	6.82	7.79	14.61	10,104	0.77	2.04	22.8
	OF_15_	0.83	5.9	6.72	8,789	0.68	5.59	7.05	12.64	10,419	0.68	1.675	19.36
	OF_30_	0.7	5.17	5.87	9,181	0.57	4.74	6.54	11.28	10,897	0.60	1.45	17.15
	OF_45_	0.62	4.51	5.12	8,430	0.53	4.24	5.77	10	9,858	0.59	1.185	15.12

Note: Σ represents cumulative. CK is no N fertilizer, single application of P and K fertilizers, CF is local farmer’s fertilization method, OF_15_ is 15% substitution of chemical fertilizer N with pig manure during wheat base fertilization, OF_30_ is 30% substitution, and OF_45_ is 45% substitution.

### 3.5 Annual total N loss, including NH_3_ and NO_3_^-^ leaching

Cumulative NH_3_ emissions and NO_3_^-^ leaching exhibited significant variations under different pig manure substitution ratios (**Table 2**). Specific data revealed that under the OF_30_ treatment, cumulative NH_3_ emissions reached 4.63 kg N ha^−1^, while NO_3_^-^ leaching was 1.40 kg N ha^−1^. In contrast, under the OF_45_ treatment, cumulative NH_3_ emissions decreased to 4.08 kg N ha^−1^, and NO_3_^-^ leaching declined to 1.23 kg N ha^−1^. These values, upon integrating annual monitoring results, demonstrated that increasing the pig manure substitution ratio significantly reduced both NH_3_ volatilization and NO_3_^-^ leaching, highlighting the effectiveness of higher substitution ratios in mitigating environmental risks. An analysis indicated a notable synergistic reduction trend between cumulative NH_3_ emissions and NO_3_^-^ leaching. The OF_45_ treatment showed an 11.80% decrease in cumulative NH_3_ emissions compared to OF_30_ and a 12.14% reduction in NO_3_^-^ leaching, reflecting the comprehensive inhibitory effect of increased pig manure substitution on nitrogen environmental losses. Disaggregated data from the wheat and maize seasons revealed that the maize season contributed more to NO_3_^-^ leaching, whereas NH_3_ volatilization was more pronounced during the wheat season. However, higher pig manure substitution ratios effectively reduced peak loss events in both seasons.

## 4 Discussion

### 4.1 Trade-off between environmental benefits and wheat–maize yields

Pig manure application can achieve a larger benefit of chemical fertilizer replacement. Its incorporation ratio should be optimum (Xu et al., 2024b). Since fertilization and N dynamics have a strong relationship (**Figure 5**), this study confirms that the OF_30_ (30% pig manure amendment) treatment effectively mitigated the imbalance between environmental N losses and yield through synergistic organic–inorganic nutrient release effects. It exhibited 22%–27% less total N loss and 22%–32% lower NH_3_ emissions per unit g kg^-1^ of wheat–maize grain along with a 6%–8% yield increase. Significant effects of manure on cumulative gaseous N loss are well documented (Hei et al., 2023). The higher substitution of chemical fertilizer with pig manure by 45% in OF_45_ maximized environmental gains, but at the cost of lower productivity, which is consistent with the findings of Niu et al. (2024) who reported that annual wheat–maize yields and sustainable yield index were maintained when the chemical fertilizer replacement ratio with manure was close to 30%. The mechanisms underlying the effects of pig manure substitution can be attributed to several factors. First, 30% pig manure substitution enhances soil pH, total C content, and acid phosphatase activities, which is proven in the study of Liu et al. (2025). This also aligns with the “threshold effect” hypothesis proposed by Zhang et al. (2023), which posits that organic substitution should not exceed 30% (Hou et al., 2025). Second, the humic acid components in pig manure may activate the expression of glutamine synthetase genes, thereby promoting crop N assimilation efficiency (Chen et al., 2025a). Moreover, this study reveals that the yield decline at 45% pig manure in the OF_45_ treatment was not solely due to insufficient N supply. The lower yields in OF_45_ compared to OF_30_ and OF_15_ could be caused instead by changes in soil properties due to excessive manure application, mainly an increase in soil pH toward a less optimal range for N mineralization, an imbalanced C/N ratio that promotes microbial immobilization and P overload, which may lead to soil nutrient antagonism and soil salinity stress. Manure substitution ratio significantly affects total and available soil nutrients (Hao et al., 2025). Considering practical fertilizer management, the OF_30_ treatment with a 30% pig manure substitution for chemical N fertilizer is highly feasible. It aligns with typical manure use in integrated crop–livestock farms and does not require complex logistics for transport and application. Generally, smallholder farmers face challenges in adopting high-rate manure application due to limited labor, infrastructure, and resources (Deng et al., 2024; Yin et al., 2025). In addition, 30% pig manure ratio helps sustain yield stability by avoiding the toxicity and salinity stress risks associated with higher application rates during long-term use. Taking into account all of these factors, the results indicated that the economic externalities of agricultural non-point source pollution control should be incorporated into technology evaluation systems. This agrees closely with the full-cost accounting concept for low-carbon agricultural technologies emphasized by the Intergovernmental Panel on Climate Change (He et al., 2023b). Overall, the results demonstrated that pig manure replacement can effectively reduce NH_3_ and NO_3_^-^ leaching losses in cropland. Hence, the findings provide empirical support to optimize N management practices in wheat–maize cropping systems.

### 4.2 Technical optimization directions and sustainability

Although OF_30_ (30% amendment of pig manure) demonstrates significant advantages, its long-term application could still face two key challenges: First, regional variations in the nutrient content of pig manure may lead to “threshold drift” in substitution ratios—for instance, the total N content of pig manure used in this experiment was 2.1%. If applied to low-quality organic composted materials (e.g., ≤1.5% N), the substitution ratio’s impact on mineralization rates must be re-evaluated. Typically, the pig manure composting process leads to significant changes in N content (Wang et al., 2024a). In addition, area-to-area soil textural differences could also impact chemical replacement ratios with pig manure and its effects on N dynamics (Norberg and Aronsson, 2024). Moreover, the availability of local manure resources and crop and soil nutrient status can be factors affecting optimal manure ratios (Sun et al., 2025). Second, the annual local precipitation variability in regions ranges from 25% to 30%, which may alter soil water movement pathways and influence NO_3_^-^ leaching risks (Long and Sun, 2012).

### 4.3 Limitations and future research

This study primarily focused on nitrogen dynamics in maize–wheat cropping systems under various substitution ratios of pig manure. It did not consider potential changes in soil physicochemical properties. The application of pig manure ratios can influence key salinity parameters such as soil pH and EC. Future research should examine how critical soil indicators fluctuate under different pig manure ratios to provide a more comprehensive environmental assessment and fully understand the trade-offs involved in chemical fertilizer substitution strategies. Additionally, it would be valuable to explore how pig manure substitution ratios affect soil P cycling. Furthermore, research should also investigate how different manures vary in nutrient content based on the feed source used at various farms. In countries where pig manure is unavailable or not acceptable (forbidden), identifying alternative animal manures that can be used at similar chemical N fertilizer substitution ratios and understanding how they impact crop yield and N loss reduction are important areas for future study.

## 5 Conclusions

This study quantitatively assessed the trade-offs between pig manure substitution, crop yield, NO_3_^-^ losses, and NH_3_ emissions in a wheat–maize rotation to identify the best strategy to reduce the negative environmental effects of synthetic fertilizers while maintaining crop productivity. The results indicated that replacing 30% of chemical N fertilizer with pig manure (OF_30_) significantly lowers NH_3_ emissions without sacrificing crop yields. Compared to conventional fertilization practices (CF), this treatment reduces total annual NH_3_ emissions by 16%–27% and decreases NO_3_^-^ leaching losses by 30%–35%. Increasing the substitution ratio of chemical N fertilizer with pig manure further reduces N losses but does not improve yields. The 30% substitution rate of pig manure is likely the best balance between crop production and environmental sustainability. These findings are valuable to reduce agricultural non-point source pollution in the region by effectively utilizing organic manure resources while boosting crop yields.

## Data Availability

The original contributions presented in the study are included in the article/supplementary material. Further inquiries can be directed to the corresponding author.
